# Differential pathogenesis of closely related 2018 Nigerian outbreak clade III Lassa virus isolates

**DOI:** 10.1371/journal.ppat.1009966

**Published:** 2021-10-11

**Authors:** Derek R. Stein, Bryce M. Warner, Jonathan Audet, Geoff Soule, Vinayakumar Siragam, Patrycja Sroga, Bryan D. Griffin, Anders Leung, Allen Grolla, Kevin Tierney, Alix Albietz, Darwyn Kobasa, Abdulmajid S. Musa, Adama Ahmad, Afolabi M. Akinpelu, Nwando Mba, Rebecca Rosenke, Dana P. Scott, Greg Saturday, Chikwe Ihekweazu, David Safronetz

**Affiliations:** 1 Zoonotic Diseases and Special Pathogens, National Microbiology Laboratory, Public Health Agency of Canada, Winnipeg, Canada; 2 Department of Medical Microbiology, University of Manitoba, Winnipeg, Canada; 3 Nigerian Centre for Disease Control, Jabi, Abuja, Nigeria; 4 Rocky Mountain Veterinary Branch, Rocky Mountain Laboratories, National Institute of Allergy and Infectious Diseases, National Institutes of Health, Hamilton Montana, United States of America; University of Pittsburgh, UNITED STATES

## Abstract

Nigeria continues to experience ever increasing annual outbreaks of Lassa fever (LF). The World Health Organization has recently declared Lassa virus (LASV) as a priority pathogen for accelerated research leading to a renewed international effort to develop relevant animal models of disease and effective countermeasures to reduce LF morbidity and mortality in endemic West African countries. A limiting factor in evaluating medical countermeasures against LF is a lack of well characterized animal models outside of those based on infection with LASV strain Josiah originating form Sierra Leone, circa 1976. Here we genetically characterize five recent LASV isolates collected from the 2018 outbreak in Nigeria. Three isolates were further evaluated *in vivo* and despite being closely related and from the same spatial / geographic region of Nigeria, only one of the three isolates proved lethal in strain 13 guinea pigs and non-human primates (NHP). Additionally, this isolate exhibited atypical pathogenesis characteristics in the NHP model, most notably respiratory failure, not commonly described in hemorrhagic cases of LF. These results suggest that there is considerable phenotypic heterogeneity in LASV infections in Nigeria, which leads to a multitude of pathogenesis characteristics that could account for differences between subclinical and lethal LF infections. Most importantly, the development of disease models using currently circulating LASV strains in West Africa are critical for the evaluation of potential vaccines and medical countermeasures.

## Author summary

Lassa fever is a severe viral hemorrhagic fever of humans caused by infection with Lassa virus, which is endemic in many countries in West Africa. Annually, an estimated 300,000–500,000 people are infected with Lassa virus, making it one of the most prominent agents responsible for hemorrhagic disease in humans. Despite this significant burden of disease, to date, no approved therapeutic or prophylactic vaccine exists for Lassa fever, due in part to a lack of characterized animal models for studying the disease. Here, we describe guinea pig and non-human primate models for Lassa fever using recently isolated viruses from a 2018 outbreak of Lassa fever in Nigeria. Despite similar collection locations and dates, the isolates obtained from human infections demonstrated a high degree of genotypic heterogeneity and phenotypic characteristics in animal models resulting in both lethal and non-lethal infections. Of interest, one isolate resulted in significant respiratory manifestations, an under-reported disease manifestation in humans. These models will provide comparative models to those already characterized and aid in elucidating disease characteristics of Lassa fever. In addition, they will serve the immediate purpose of evaluating known and novel medical countermeasures to treat and prevent disease in West Africa.

## Introduction

Lassa virus (LASV) is an etiological agent of hemorrhagic fever, referred to as Lassa fever (LF) endemic to some parts of Western African countries of Nigeria, Guinea, Sierra Leone, and Liberia [1,2]. In 2018, Nigeria experienced a large LASV outbreak, with over 3498 suspected and 633 confirmed positive cases. The outbreak resulted in a total of 171 deaths with a 27% case fatality rate. The overall disease burden in Nigeria has been steadily increasing since 2017 with recurring seasonal outbreaks [3–6]. In terms of global disease burden, LF is one of the most prevalent hemorrhagic fevers in humans with an estimated 300,000–500,000 infections annually [2,7,8]. Transmission is mainly driven through peridomestic rodents and in particular the natal multimammate rat (*Mastomys natalensis*) [2,7–9]. It remains unclear if the increased severity and frequency of outbreaks in countries like Nigeria is due to climatic changes impacting the ecology of the virus/rodent reservoirs or enhanced detection.

LF has an incubation period of between 1 and 3 weeks, and typically presents with fever, headache, diarrhea, vomiting, elevated liver enzymes, elevated hematocrit, and muscle/joint pain. Interestingly, LF can manifest in humans from mild asymptomatic infection to severe hemorrhagic fever with multi-organ failure [3,10,11]. It is estimated that 1–2% of cases represent this severe phenotype, however in outbreak scenarios the case fatality rate has been documented to reach 30% [11,12]. The majority of LF signs observed in humans signs are recapitulated in the non-human primate (NHP) model, and the inbred (strain 13) guinea pig model may also include severe respiratory dysfunction associated with interstitial pneumonia although with limited necrotizing hepatitis [13–15].

Complicating matters, LASV has a high degree of genetic diversity that correlates with geographic distribution, making our current knowledge describing the breadth of pathogenesis severely lacking [16,17]. Recent studies have highlighted a larger spectrum of LASV pathological signs through *in vivo* comparisons of isolates from Sierra Leone (Josiah), Liberia (Z-132) and Mali (Sormoba-R) [18]. Animals infected with the Malian isolate presented with severe pulmonary manifestations and delayed disease progression resulting in reduced mortality in NHPs. The majority of pathogenesis and vaccine studies have been conducted using the Clade IV Sierra Leone isolate, Josiah [19–22]. The characterization of genetically and geographically diverse LASV isolates in animal models of disease is imperative to understanding the scope of LASV pathogenesis and the possible discovery of a universal vaccine candidate that can be deployed throughout West Africa. Here we describe the use of both strain 13 guinea pigs and cynomolgus macaques to establish and characterize lethal models of LASV infection using contemporary isolates from the 2018 Nigerian outbreak. Notably, we describe two isolates with atypical disease manifestations despite originating from the same geographical region and time period.

## Results

### Virus isolation

Five serum samples were collected during the Nigerian outbreak in February 2018 from the provinces of Nasarawa and Plateau, north of the Niger River where the majority of infections have been identified as Clade III viruses [23]. Specifically, samples were from one male and one female, NML-33 and NML-55 respectively, from Nasarawa State, two females, NML-46 and NML-61, from Plateau State, and one female, NML-57, from an unknown location. Samples were collected during symptomatic illness and confirmed LASV positive by RT-PCR by the Nigerian Centre for Disease Control. Patient outcomes were as follows: NML-33, -46, and -55 were from fatal cases, NML-61 was non-lethal and NML-57 had an unknown outcome. Serum samples were sent to a Biosafety level 4 (BSL 4) laboratory at the National Microbiology Laboratory of the Public Health Agency of Canada in accordance with international regulations and under approved Nigerian export and Canadian import permits. Virus was isolated in Vero cell culture according to standard protocols. Cytopathic effect was visible at 7 days post-infection (dpi) and titers ranged from 10^4^ to 10^7^ 50% tissue culture infectious doses (TCID_50_) per mL.

### Phylogenetic analysis of Nigerian outbreak isolates

**Fig 1:**
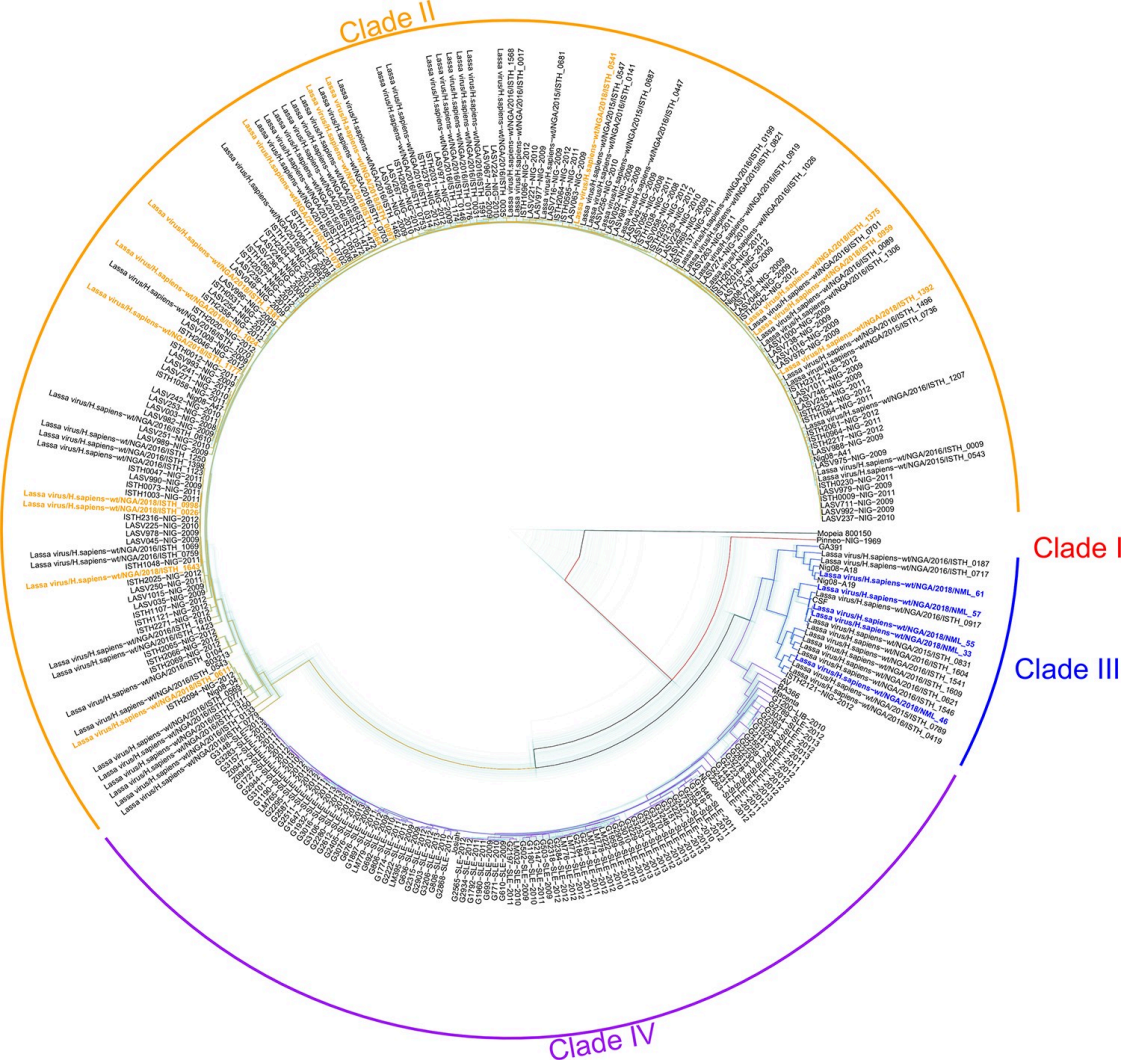
Phylogenetics of five 2018 Nigerian outbreak Lassa virus isolates. BEAST 2.5.1 was used to produce a time-aware phylogeny of the full S segment of 287 LASV isolates, in addition to the 5 viruses sequenced in this study. Mopeia virus strain AN 21366 was used to root the tree. The light blue trees show 101 posterior draws, displaying the linkage and divergence uncertainty. The main (colored) tree is the median tree, as calculated using TreeAnnotator. The isolates sequenced in this study are highlighted in blue (Clade III), other contemporary isolates (Nigeria, 2018) are highlighted in orange.

The five p1 isolates were subjected to next-generation sequencing for phylogenetic analysis (S segment accession numbers NML-33 MZ169791, NML-46 MZ169793, NML-55 MZ169795, NML-57 MZ169797, NML-61 MZ169799; L segment accession numbers NML-33 MZ169790, NML-46 MZ169792, NML-55 MZ169794, NML-57 MZ169796, NML-61 MZ169798). All five isolates were identified as Clade III based on S segment phylogenetic alignment confirming the geographic origin of the cases (Fig 1, Table 1). The isolates from Nasarawa State (NML-33 and -55) are very closely related to each other. On the other hand, the isolates from the Plateau State (NML-46 and -61) are highly divergent, being only related through the common ancestor of all Clade III viruses. NML-46 also shares a more recent ancestor with the Nasarawa State isolates than with NML-61. The isolate from an unknown region appears somewhat close to the ones from Nasarawa State.

**Table 1:** Percent identity of the Small genomic segments of the Nigerian Lassa virus isolates to reference Lassa virus sequences.

	LASV-Pineo	LASV-Josiah	NML-33	NML-46	NML-55	NML-57	NML-61
LASV-Pineo		76.2	76.2	76.2	72.0	75.9	74.7
LASV-Josiah	29.3		78.5	79.4	74.3	77.5	77.0
NML-33	29.2	26.1		83.7	97.0	84.9	79.2
NML-46	29.2	24.6	19.3		81.2	84.4	80.0
NML-55	29.8	26.5	0.3	19.7		80.8	75.7
NML-57	29.3	27.3	17.6	18.3	17.4		78.0
NML-61	30.4	27.0	25.0	23.8	25.0	26.0	

% similarity (upper boxes) and % divergence (lower boxes) of the five novel Nigerian clade III isolates to Pineo and Josiah reference sequences. Accession numbers: Josiah (AY628203.1), Pineo (KM822128.1), NML-33 (MZ169791), NML-46 (MZ169793), NML-55 (MZ169795), NML-57 (MZ169797), NML-61 (MZ169799).

### Disease progression and pathogenesis in strain 13 guinea pigs

**Fig 2:**
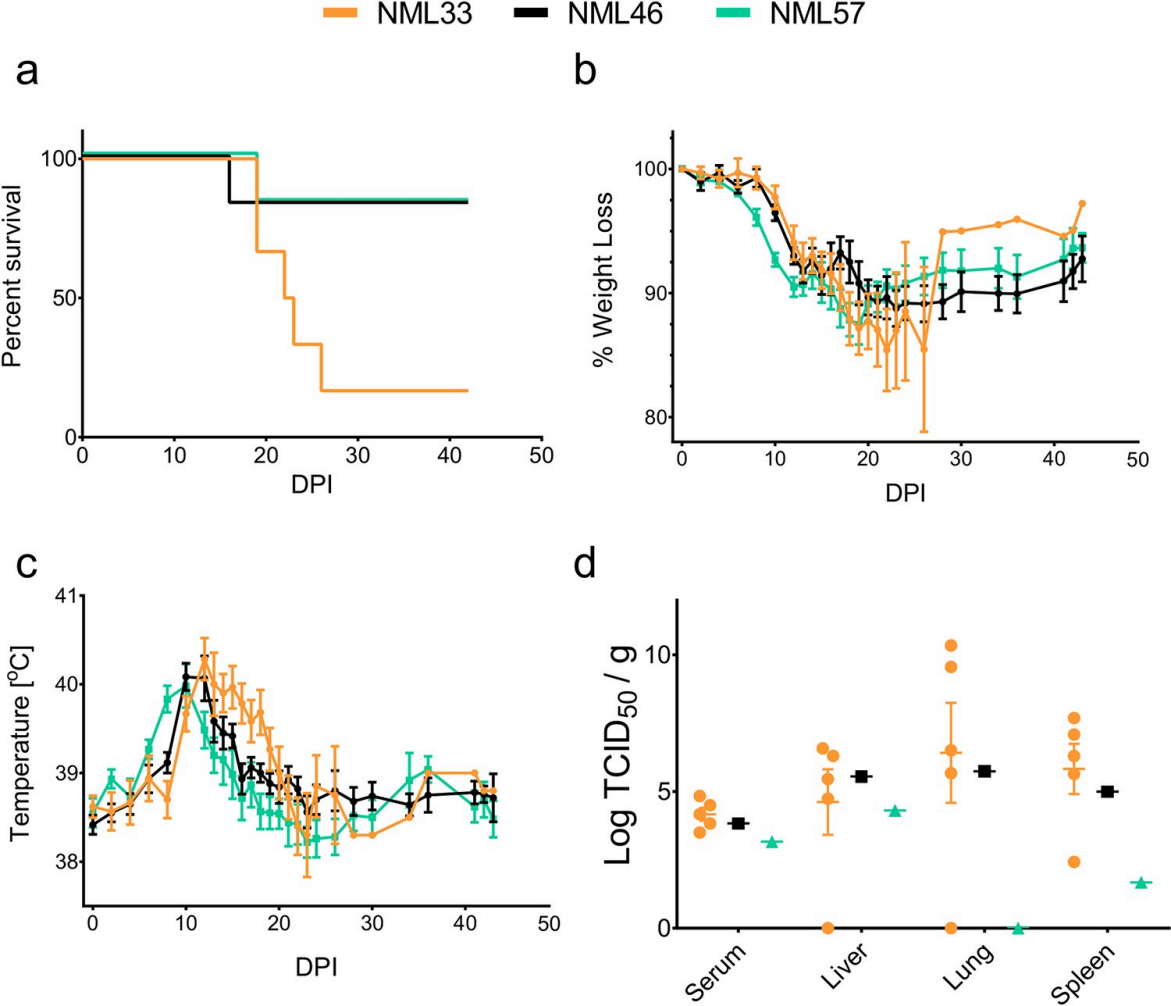
Infection of strain 13 guinea pigs with three 2018 Nigerian outbreak Lassa virus isolates. Groups of 6 guinea pigs (2 female, 4 males) were infected with 1x10^4^ TCID_50_ of each Nigerian outbreak isolate; NML-33 (orange), NML-46 (black), NML-57 (green) and measured for signs of disease including survival (a), weight loss (b), and temperature (c). Serum and tissues (liver, lung, and spleen) were also collected from moribund animals and assayed for infectious virus (d). Data are presented as mean values with error bars indicating SEM.

Three LASV isolates (NML-33, -46 and, -57) were selected to characterize in the strain 13 guinea pig model. Groups of six guinea pigs (2 female, 4 male) were infected with 1x10^4^ TCID_50_ by the intraperitoneal route for each isolate and monitored for signs of LASV disease for up to 42 days. Although signs of disease varied slightly with respect to onset, no obvious differences were recorded across the groups with little discrepancy from previous studies [18], particularly in animals that progressed towards lethal disease. Despite showing early signs of disease only a single guinea pig infected with the NML-57 isolate succumbed to infection at 19 dpi (Fig 2A). Guinea pigs infected with the NML-57 isolate began to show signs of disease earlier in comparison to the other two isolates with increased temperatures and weight loss noted at 7 and 8 dpi, respectively (Fig 2B and 2C). Animals infected with NML-46 and NML-33 began to lose weight on day 10 post-infection along with a spike in body temperature. Animals infected with NML-46 and NML-57 reached peak fevers of 40.1°C (12 dpi) and 40.0°C (10 dpi), respectively. A single animal in each of the NML-46 and NML-57 infection groups continued to lose weight following peak temperature, developed increased respiration and hypothermia requiring euthanasia on days 16 and 19 post-infection, respectively. NML-33 was considerably more lethal than NML-46 and NML-57 with five of six guinea pigs succumbing to infection with an average time to death of 21.8 days (range of 19–26 days; Fig 2A). The NML-33-infected guinea pigs reached a peak temperature of 40.3°C at 12 dpi. Several animals began to show signs of increased respiration by 13 dpi which coincided with hunched posture. While peak temperatures began to subside over the following 10 days, animals continued to lose weight with worsening respiratory indicators (rapid, shallow abdominal breathing) and hypothermia, requiring euthanasia. Samples were collected from moribund animals in order to assess infectious virus in the serum, liver, lung, and spleen (Fig 2D). Infectious virus in the serum of NML-33-infected animals reached an average of 4.2 Log_10_ TCID_50_/ml while NML-46 and NML-57-infected animals had 3.8 and 3.2 Log_10_ TCID_50_/ml, respectively. Higher levels of infectious virus were isolated in the tissues, with the lung reaching an average of 6.4 Log_10_ TCID_50_/mg in NML-33 infected animals. Samples collected from NML-33-infected animals had slightly higher levels of virus overall compared to the other two isolates (NML-46 and NML-57); however, among animals that succumbed to disease, there were similar levels of viral titers observed with no statistically significant differences in tissue types and serum collected at the time of euthanasia. All animals surviving to the end of the study (42 days) seroconverted to the LASV nucleoprotein. Infectious virus was not isolated from survivors at 42 dpi. Isolate NML-57’s rapid onset of increased temperatures and weight loss compared to the other two isolates as well as the uniform pathogenesis and high mortality rates observed in both males and females infected with NML-33, led us to further characterize these two isolates in a NHP model.

### Pathogenesis of Nigerian outbreak isolates in cynomolgus macaques

**Fig 3:**
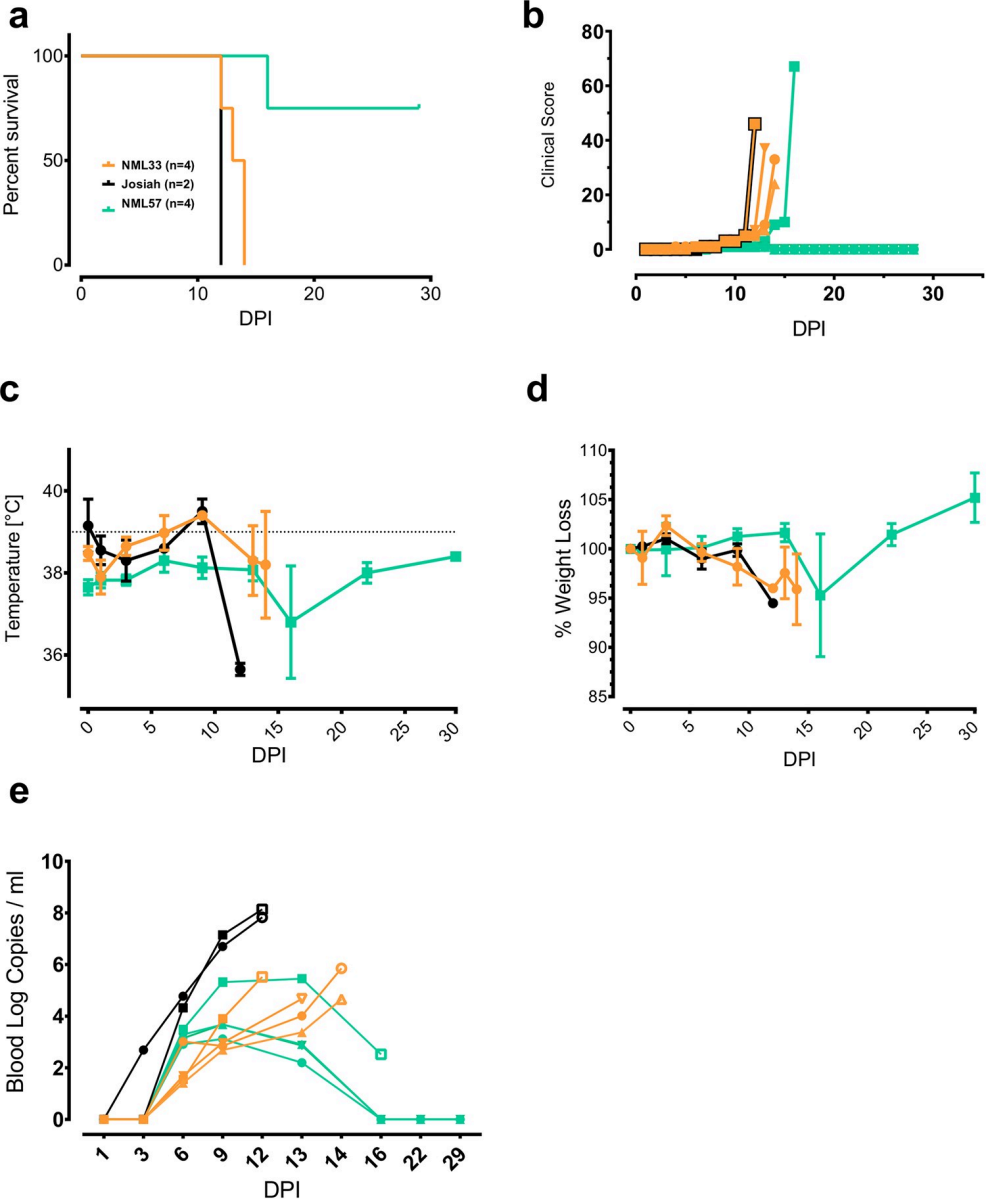
Infection of cynomolgus macaques with 2018 Nigerian outbreak Lassa virus isolates. Groups of 4 female macaques were infected with 1x10^4^ TCID_50_ of each Nigerian outbreak isolate; NML-33 (orange) and NML-57 (green) along with 2 control animals (Josiah; black) and measured for signs of disease including survival (a), clinical score (b), temperature (c), and weight loss (d). Quantities of viral RNA (copies/ml) was monitored every three days by qRT-PCR (e). Data are presented as mean values with error bars indicated SEM.

**Fig 4:**
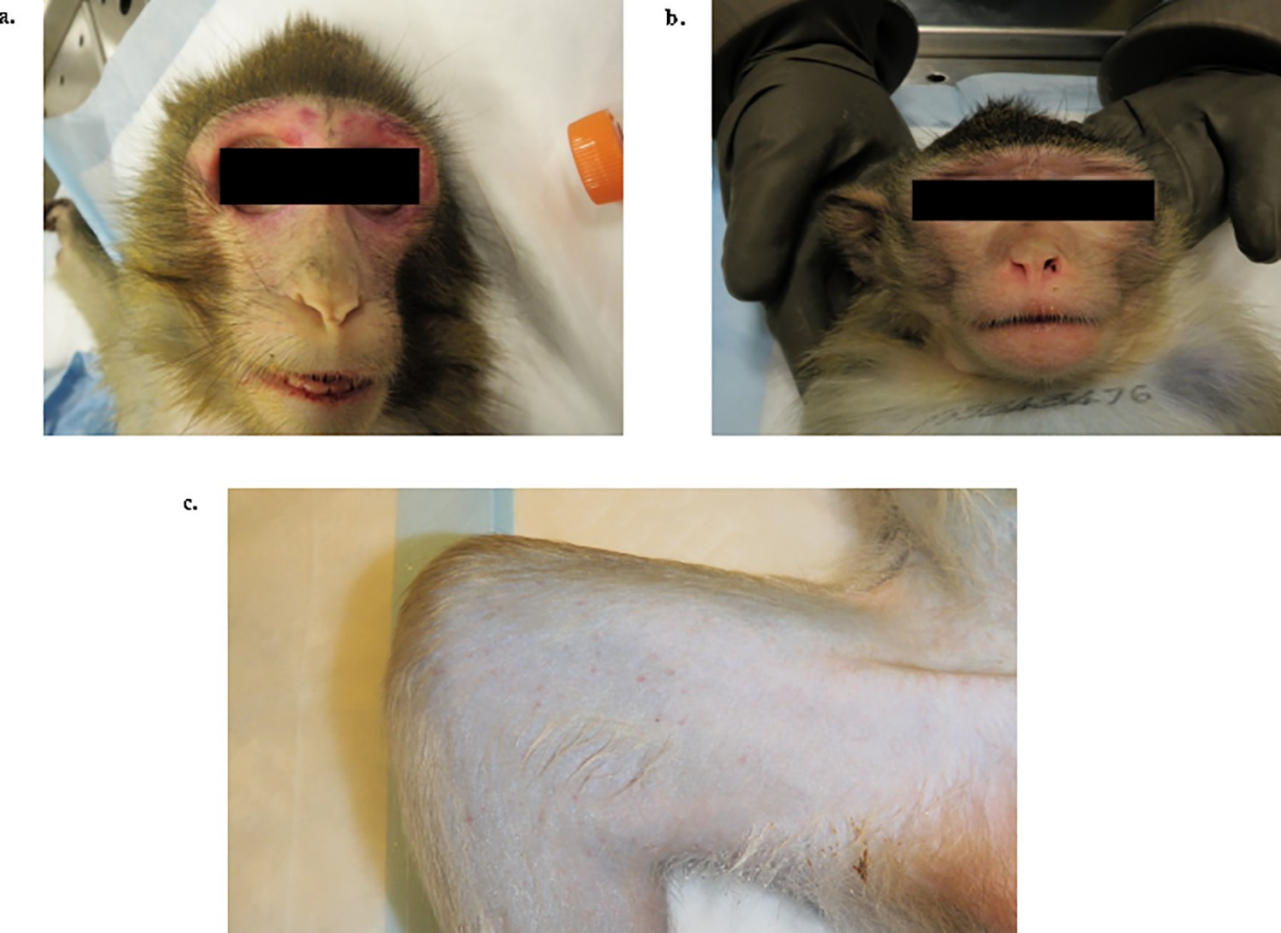
Physical appearances of macaques with a terminal 2018 Nigerian Lassa virus clade III isolate (NML-33). Perimortem observations included severe facial swelling (a), epistaxis (b), and mild petechiae (c) in NHPs infected with the 2018 NML-33 Nigerian Lassa virus clade III isolate.

Ten female cynomolgus macaques were infected with either 1x10^4^ TCID_50_ of LASV Josiah (n = 2), or one of two Nigerian isolates, NML-33 (n = 4) and NML-57 (n = 4) by the intramuscular route and monitored twice daily for signs of disease. The first notable change was recorded at 6 dpi with decreased food and water intake (25%) in animals infected with Josiah and NML-33 (Fig 3B). Increased body temperatures were noted 9 dpi in both Josiah and NML-33-infected animals with a mean of 39.4°C (peak temperature, 39.8°C) (Fig 3C). In contrast none of the NML-57-infected animals demonstrated increases in body temperature despite a single animal eventually succumbing to LASV disease. Clinical scores began to rise 9 dpi which culminated with an average weight loss of 5% in moribund animals (Fig 3D). Three out of the four NML-33-infected animals exhibited a marked increase in respiration (40, 54 and 60 breaths per minute) with one animal progressing to severe respiratory failure on day 13 post-infection requiring euthanasia. None of the animals infected with Josiah or NML-57 had noticeable respiratory signs throughout the experiment. The two Josiah-infected animals succumbed to infection at 12 dpi while the NML-33-infected animals had a slight delay in disease manifestations, succumbing to infection between 12 and 14 dpi (average: 13.25 days) (Fig 3A). At the time of euthanasia NML-33-infected animals exhibited hematochezia, epistaxis, mild petechiae, and one animal had notably severe facial swelling (Fig 4). Similar to the high lethality rate in guinea pigs, NML-33 was 100% lethal in macaques, whereas infection with NML-57 was lethal in only a single animal with a delayed time to death of 16 dpi.

### Viral burden and gross pathological observations

**Fig 5:**
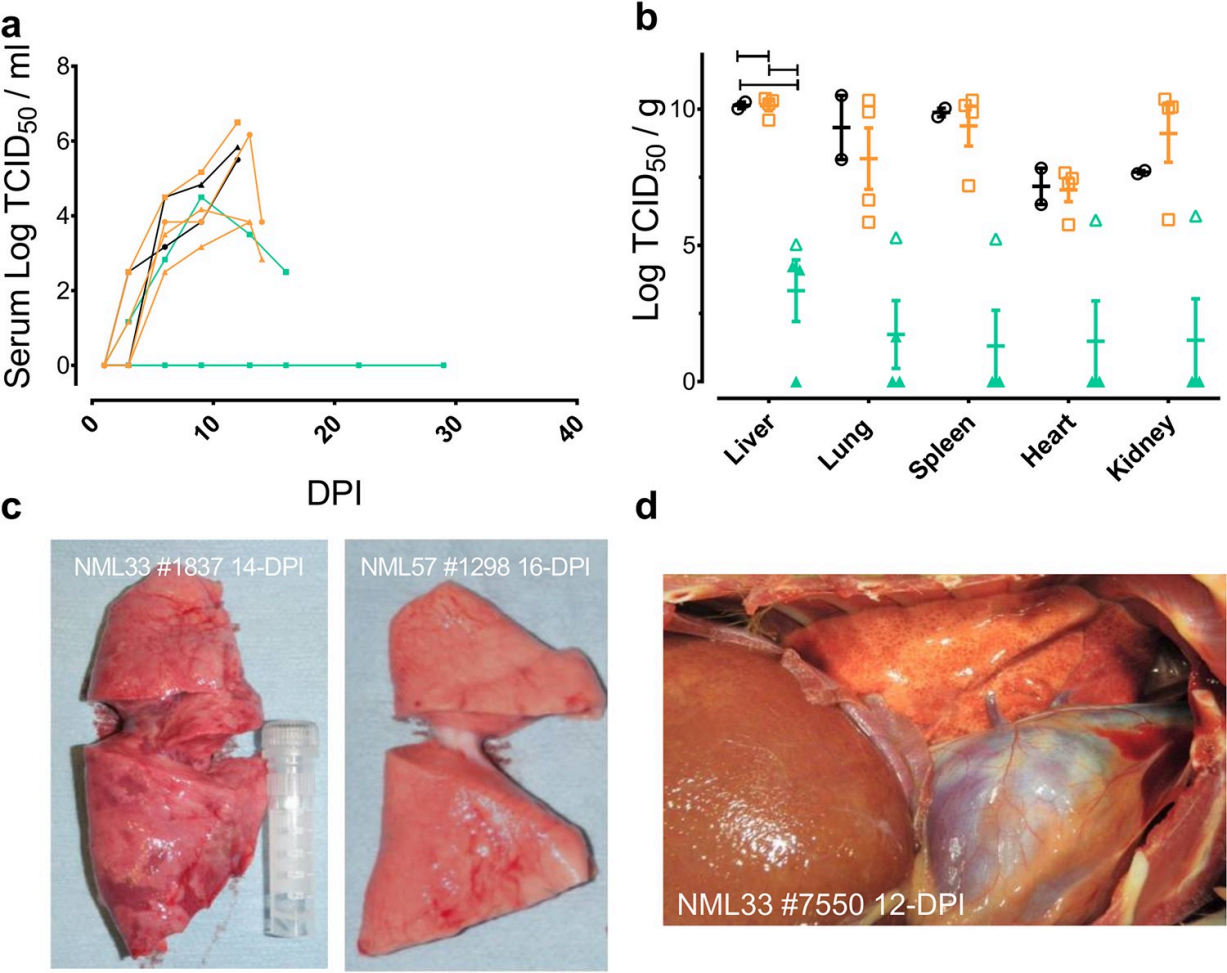
Infectious virus and gross pathological findings in Nigerian Lassa virus infected macaques. Infectious virus was assayed in the serum of infected macaques every three days (a) as well as from moribund (open circle) and surviving animals (closed circle) (b); NML-33 (orange), NML-57 (green), Josiah (black). NML-33 animals exhibited severe respiratory symptoms compared to NML-57 with significant pulmonary lesions and hemorrhage (c) and (d). Data are presented as mean values with error bars indicated SEM.

We examined viral burden in the blood and/or serum of infected animals by both RT-qPCR as well as virus titrations (TCID_50_) assays. By day 3 post-infection most animals had detectable viral RNA in serum and by day 6, infectious virus in the serum, which continued to rise to peak levels between days 12 and 13 post-infection (Figs 3E and 5A). Josiah-infected animals reached a mean titer of 5.7 Log_10_ TCID_50_/ml while NML-33-infected animals reached a peak mean titer of 4.8 Log_10_ TCID_50_/ml. Despite detectable LASV RNA in all four NML-57-infected animals (Fig 3D), only a single animal had detectable infectious virus in the serum, which reached a peak titer of 4.5 Log_10_ TCID_50_/ml on day 9 post-infection (Fig 5A).

Tissue specimens collected from moribund NHPs demonstrated systemic spread of LASV infection. Virus was isolated from the liver of the three survivors in the NML-57-infection groups as well as in the lungs of two animals (Fig 5B) at the conclusion of the study. The single NML-57 animal which ultimately succumbed to infection had detectable virus across all the tissues and had significantly less infectious virus overall compared to the NML-33 or Josiah-infected animals. When comparing the NML-33 and Josiah infected animals, both had comparable amounts of virus isolated from the liver, lung, spleen, and heart.

Consistent with the severe respiratory signs observed in NML-33 infected animals, gross pathological differences were observed in the lungs when comparing animals that succumbed to NML-33 and NML-57 LASV infection (Fig 5C and 5D). During necropsy severe pulmonary lesions and hemorrhage were observed in all NML-33 animals with significant pulmonary infiltrate, whereas the NML-57 and Josiah infected animals showed little to no signs of pulmonary involvement. In addition, the most common gross pathological findings observed across all the animals that succumbed to LASV infection included moderate hepatomegaly and splenomegaly.

### Histopathology

**Fig 6:**
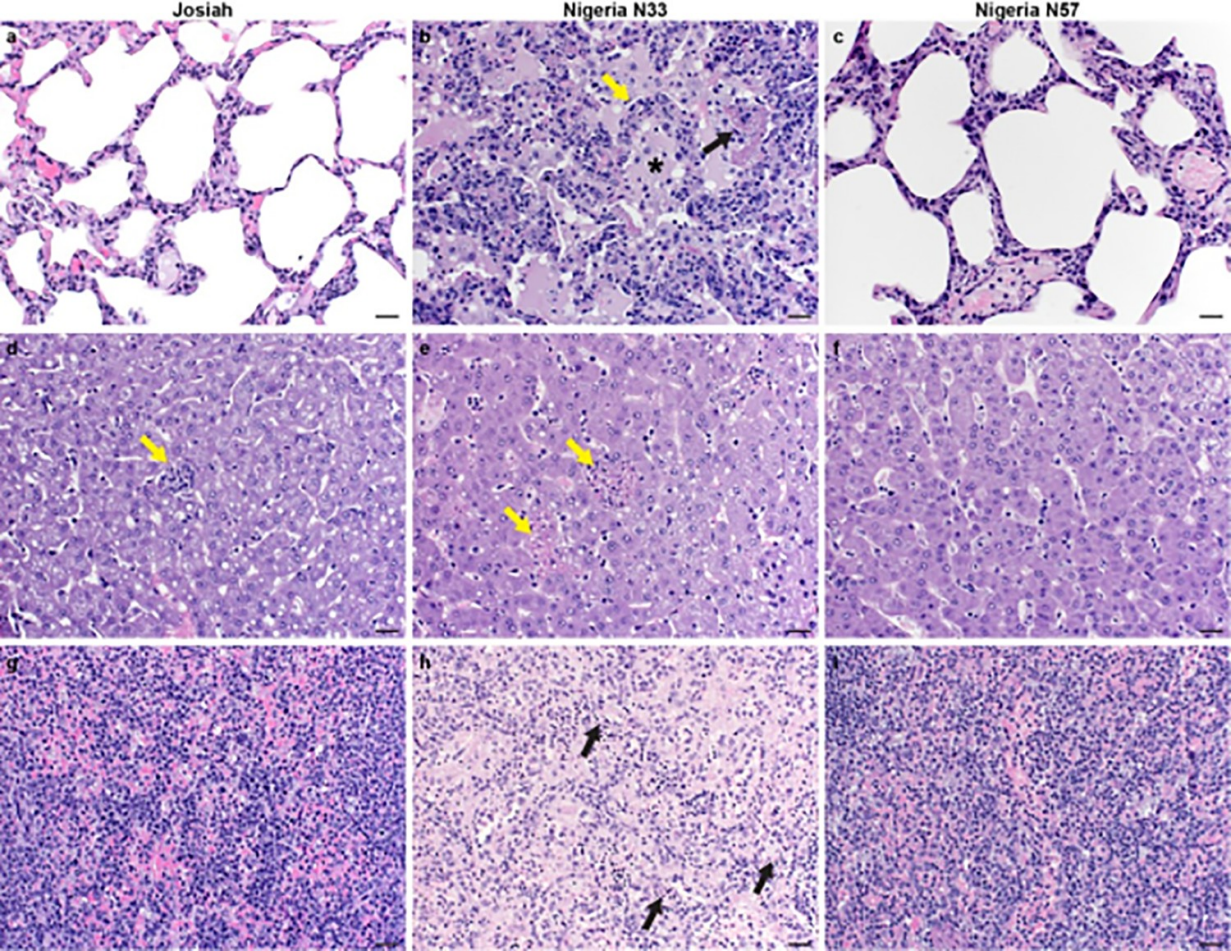
Histopathology of lung, liver and splenic lesions. Thin sections of formalin fixed tissue specimens collected at the time of severe disease from NHPs infected with Lassa virus Josiah, NML-33 and NML-57 isolates were prepared and stained using standard histopathological methods (Hematoxylin & Eosin, 200X bar = 20μm). Specimens from representative animals demonstrated **a.** Lung—Josiah—normal **b.** Lung—NML-33—intestinal pneumonia: alveolar septa expanded by edema, fibrin and inflammatory cells (yellow arrow) (fibrin = black arrow) (asterisk = edema) **c.** Lung—NML-57**—**normal **d.** Liver—Josiah—focal minimal fibrin deposits and neutrophilic infiltrates (yellow arrow) **e.** Liver—NML-33—multifocal fibrin deposits with neutrophilic histiocytic infiltrates (yellow arrows) **f.** Liver -NML-57—normal **g.** Spleen—Josiah—normal **h.** Spleen NML-33—multifocal red pulp necrosis (black arrows) **i.** Spleen—NML-57—normal.

The most striking histopathological observation was the extent of lung pathology in the NML-33 infected animals compared to Josiah and NML-57 which correlated with necropsy findings (Fig 6). Three of four animals in the Nigerian NML-33 group had mild to marked interstitial pneumonia with edema. Interstitial pneumonia was not evident in either the Josiah animals or Nigerian NML-57 animals; however, mild pulmonary edema was evident in one animal in the Nigerian NML-57 infected group that was necropsied 16 dpi. Histologically, LASV infection in the Nigerian NML-33 group resulted in mild to marked subacute interstitial pneumonia characterized by multifocal thickening of alveolar septa by edema, fibrin deposition, and varying numbers of lymphocytes, macrophages and neutrophils and multifocal type-2 pneumocyte hyperplasia. Alveoli contained moderate amounts of fibrin, edema, foamy macrophages and moderate amounts of perivascular edema. Hepatic pathology was more severe in the NML-33 group and consisted of multifocal fibrin deposits in all animals and 3 of 4 containing neutrophilic histiocytic infiltrates. One of two animals in the Josiah group had minimal fibrin deposits and neutrophilic infiltrates while inflammation was not present in the NML-57 group. Splenic red pulp necrosis was identified in 2 of 4 animals in the NML-33 group with 3 of 4 demonstrating splenic fibrin deposits. The spleens in the Josiah and NML-57 groups were essentially normal. All other lesions noted in the remaining tissues were incidental findings and are not considered to be clinically significant.

### Biochemistry, coagulation, and hematology

**Fig 7:**
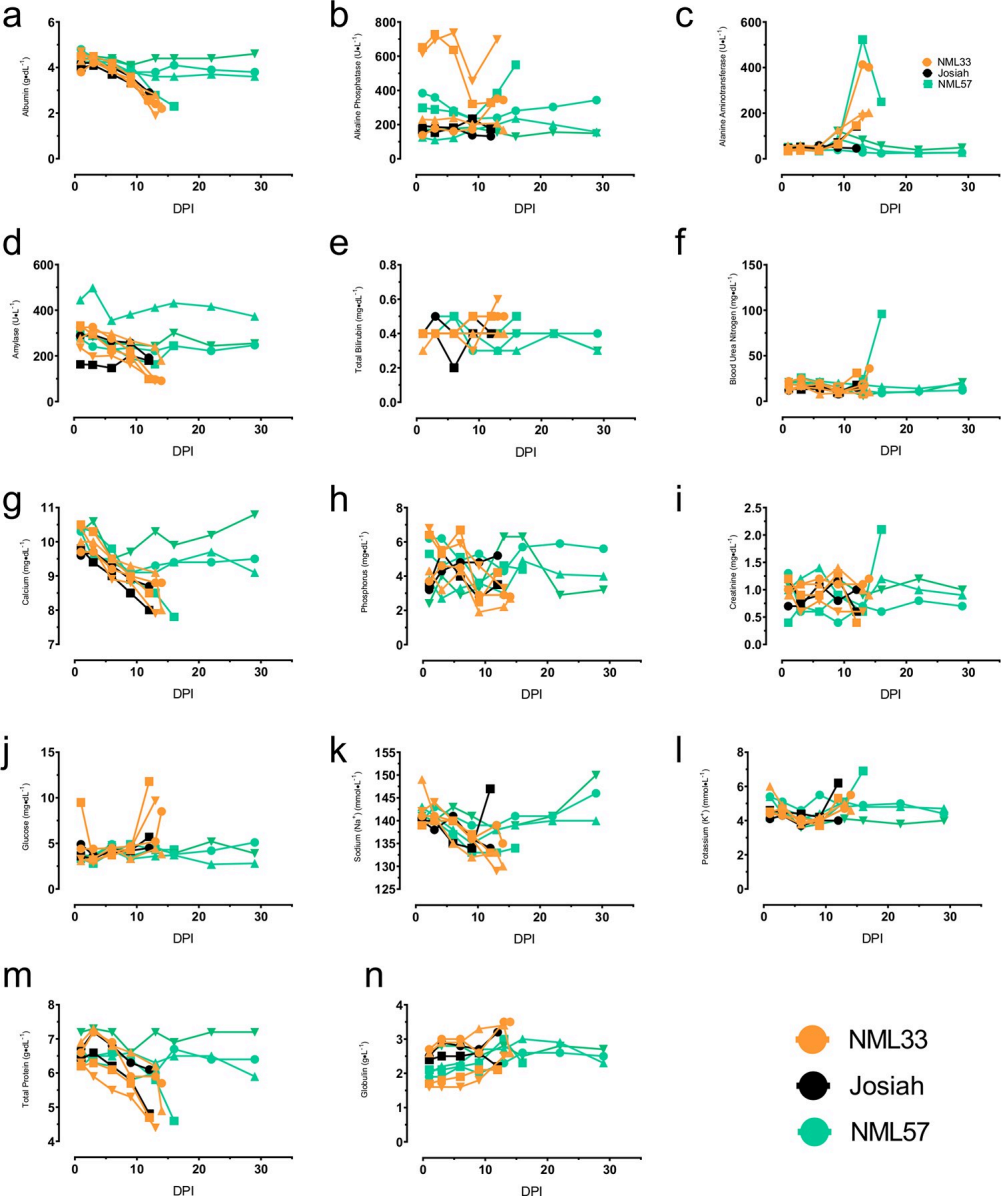
Biochemical parameters following Nigerian Lassa virus challenge. EDTA treated blood samples were collected from NHPs immediately prior to (0 dpi) and every three days post-Lassa virus infection and monitored for albumin (a), alkaline phosphatase (b), alanine aminotransferase (c), amylase (d), total bilirubin (e), blood urea nitrogen (f), calcium (g), phosphorus (h), creatinine (i), glucose (j), sodium (k), potassium (l), total protein (m), and globulin (n). Data are represented as individual data points for each animal and time point.

Liver indices were generally increased towards the end stage of infection. Alanine aminotransferase (ALT) levels began to increase by day 10 post-infection in all but one animal (Josiah) showing signs of LASV disease (Fig 7C). ALT levels peaked in all the NML-33 animals as well as a single NML-57 infected macaque between days 9–16 post-infection. Alkaline phosphatase (ALP) levels were also increased in some but not all NML-33 and NML-57 animals (Fig 7B). Both the levels of albumin and total protein decreased steadily in all animals that eventually succumbed to disease irrespective of LASV isolate (Fig 7A and 7M). All animals that went on to succumb to LASV infection trended towards hypocalcemia (Fig 7G). Despite this trend, 3 out of 4 animals infected with NML-33 became hyperglycemic just prior to succumbing to disease while all other animals remained stable throughout the experiment (Fig 7J). Both sodium and potassium levels remained relatively unchanged (Fig 7K and 7L). Of interest, a single animal infected with NML-57 had increased levels of both blood urea nitrogen (BUN) and creatinine in the terminal stage of disease indicating the onset of kidney and liver injury or failure while all other animals remained stable (Fig 7F). No discernable changes were observed in other parameters monitored.

**Fig 8:**
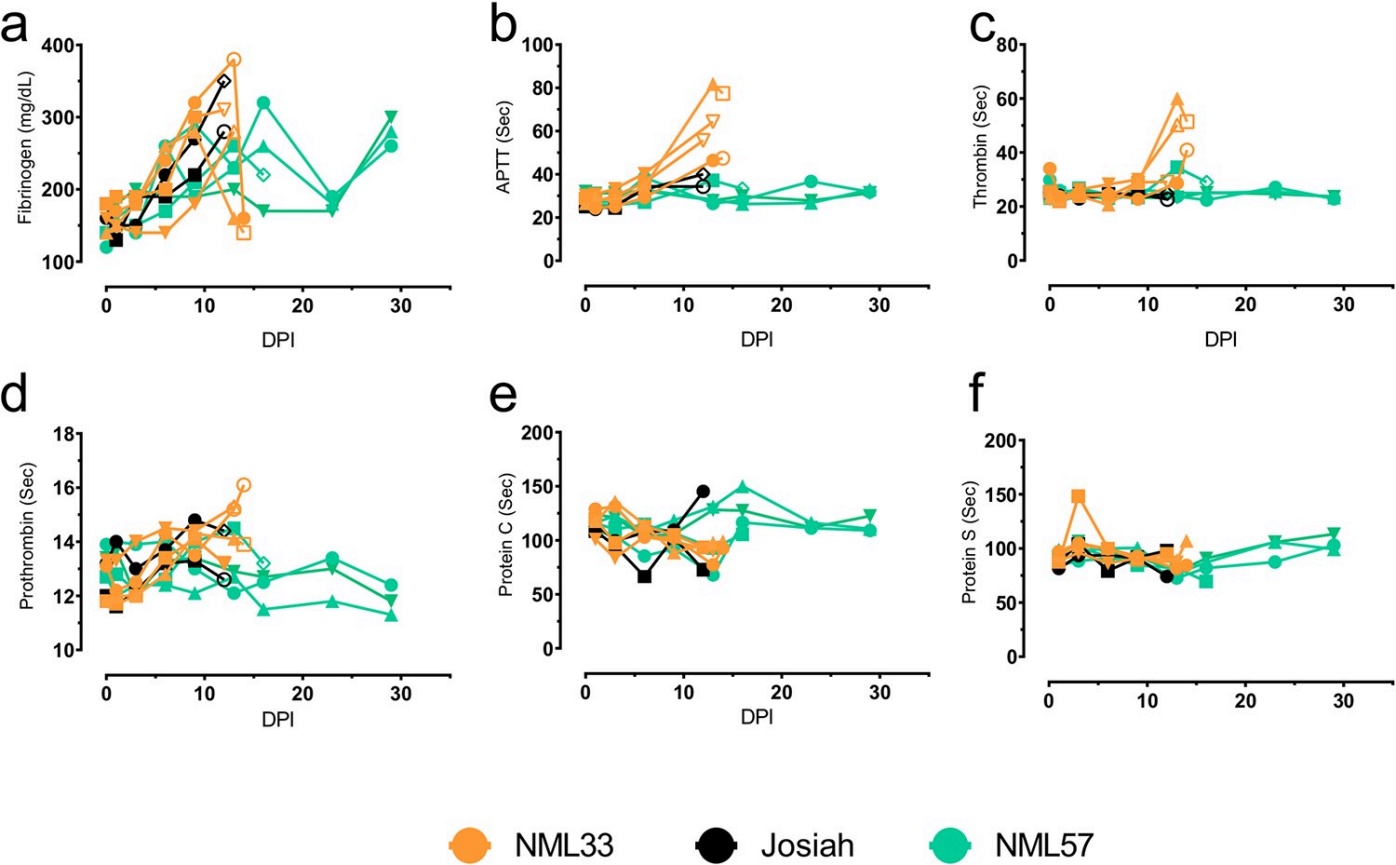
Coagulation profiles following Lassa virus infection of macaques. Citrate plasma were obtained from NHP blood samples collected every three days post-Lassa virus infection and monitored for Fibrinogen (a), aPTT (b), Thrombin (c), Prothrombin (d), Protein C (e), and Protein S (d).

Fibrinogen levels steadily increased after LASV infection in all animals, with a precipitous drop just prior to death in two of the NML-33 animals (Fig 8A). The activated partial thromboplastin time (APTT), and thrombin time of the NML-33 animals spiked significantly between day 9–14 post-infection where the Josiah and NML-57 infected animals did not show any notable trends (Fig 8B and 8C). The excessive amounts of thrombin, thrombocytopenia, elevated prothrombin (PT) levels, and rapid drop in fibrinogen just prior to death in the NML-33 animals is indicative of rapid disseminated intravascular coagulation (DIC). The development of epistaxis and mild petechiae, also supports this conclusion. There was no obvious trend in Protein S, however there was a trend towards lower protein C in NML-33 animals consistent with DIC (Fig 8D and 8E).

**Fig 9:**
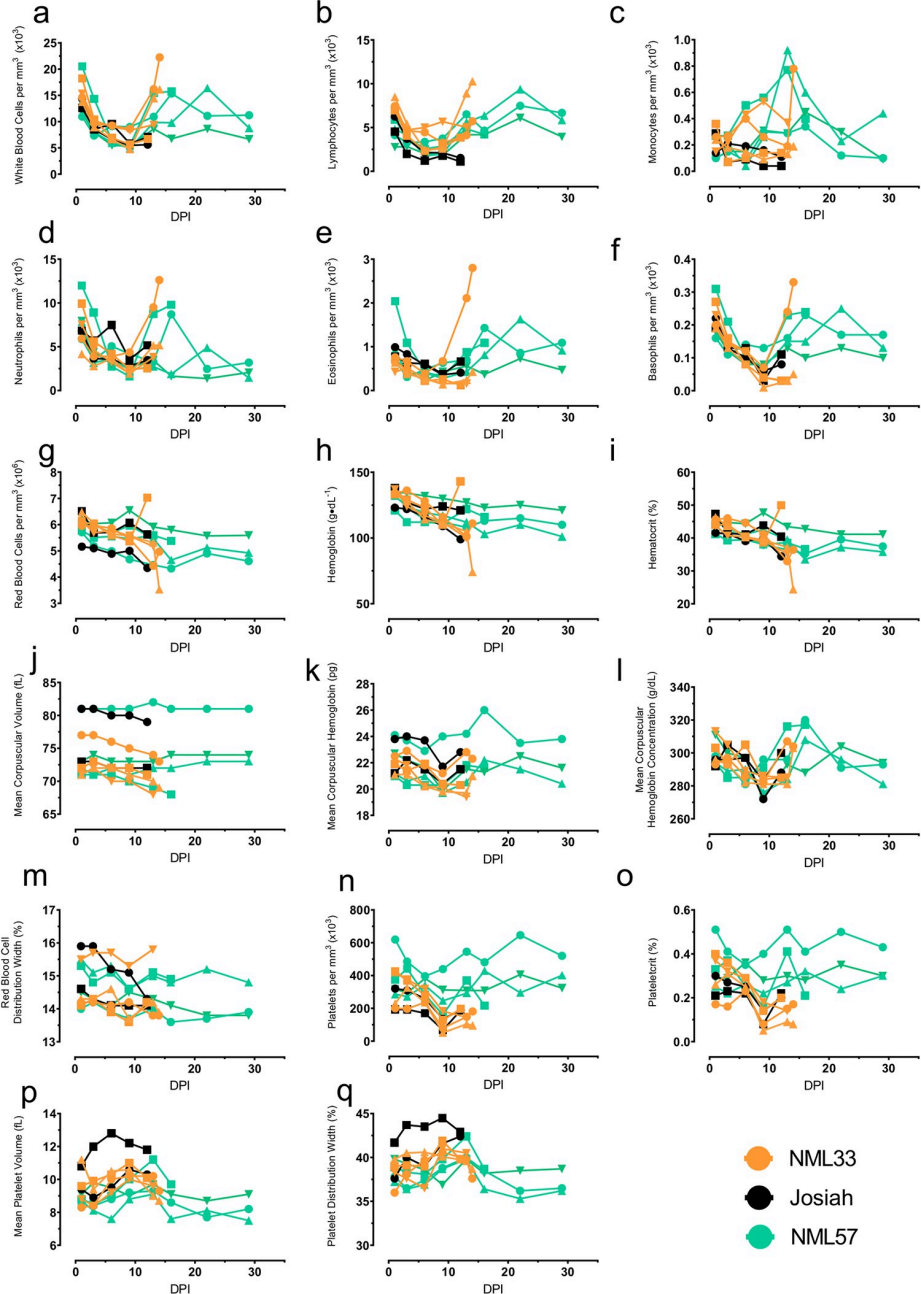
Hematological profiles in macaques following Lassa virus infection. EDTA treated blood samples were collected from NHPs every three days post-Lassa virus infection and monitored for white blood cells (a), lymphocytes (b), monocytes (c), neutrophils (d), eosinophils (e), basophils (f), red blood cells (g), hemoglobin (h), hematocrit (i), mean corpuscular volume (j), mean corpuscular hemoglobin (k), mean corpuscular hemoglobin volume (l), red blood cell distribution width (m), platelets (n), plateletcrit (o), mean platelet volume (p), and platelet distribution width (q). Data are represented as individual data points for each animal and time point.

Hematological findings were similar to previous LASV pathogenesis studies and for the most part indistinguishable between LASV isolates that caused lethal disease in our study. All animals, including the less lethal NML-57 isolate demonstrated transient lymphopenia, neutropenia, eosinopenia, and basopenia 3–10 days after infection (Fig 9). All animals had a similar decreasing trend in hemoglobin and hematocrit levels indicating hemorrhage as opposed to hemoconcentration. Leukocytosis was observed in both the NML-33 and NML-57 isolates in the late stages of disease onset due to neutropenia (12–16 dpi), while Josiah infected animals continued to trend towards leukopenia (Fig 9). Platelets levels indicated a downward trend in all moribund animals with the NML-33 infected animals having the lowest levels characteristic of thrombocytopenia. The mean platelet volume also increased during the acute phase of infection indicating consumptive platelet loss rather than decreased production (Fig 9).

### Immune cell and cytokine responses to LASV infection

**Fig 10:**
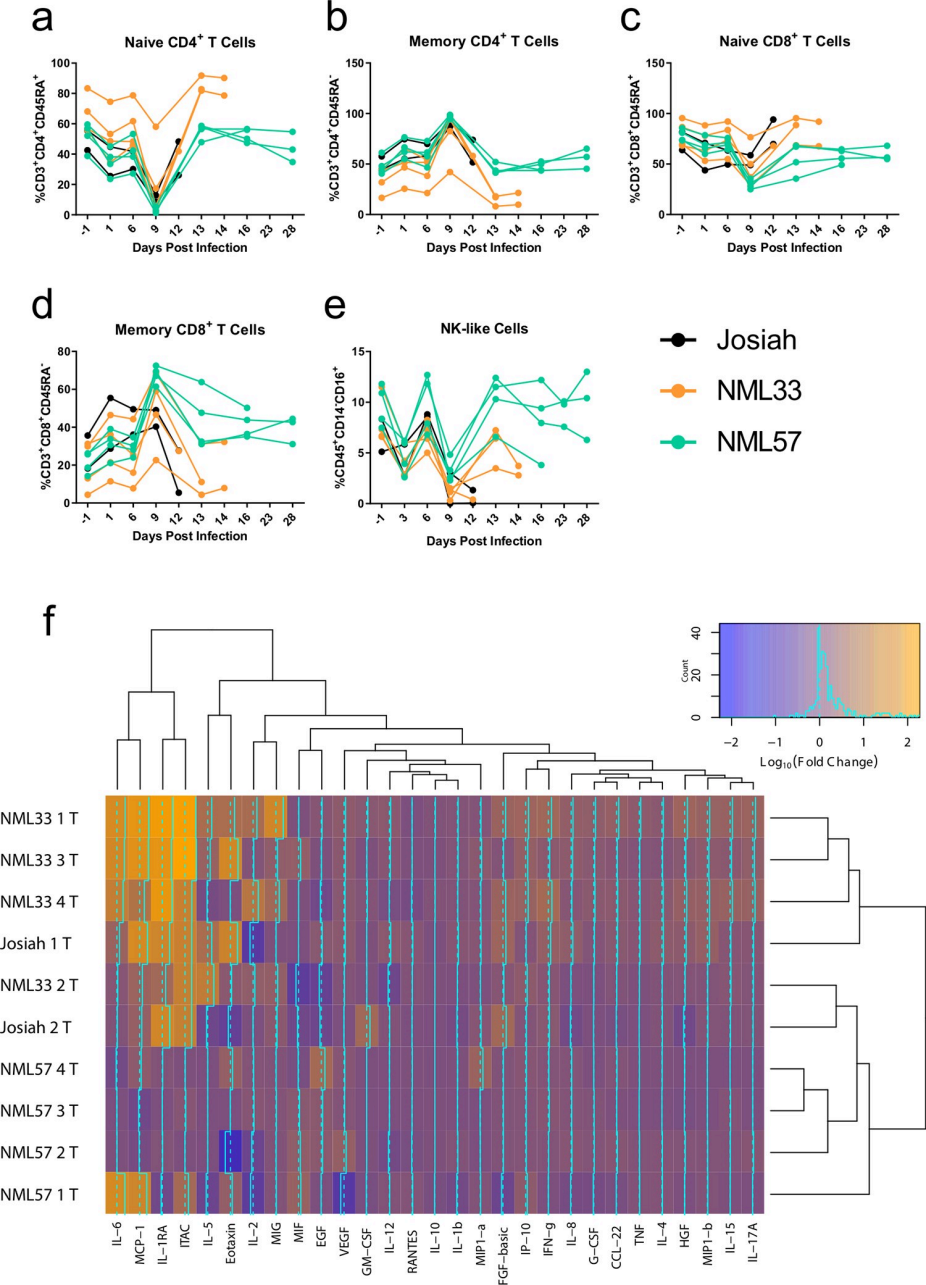
Determination of immune cell, cytokine and chemokine profile during Lassa virus infection of macaques. Flow cytometric analysis was conducted on each individual NHP at their respective time point (lines represent means for each group). Naïve CD4^+^ (a) and CD8^+^ (d) cells, memory CD4^+^ (b) and CD8^+^ (e) cells, T-regulatory cells (c), and Natural Killer cells (f) are shown. Serum was collected from LASV-infected NHPs and a Luminex assay detecting 29 analytes was performed on each sample. A heat map showing the fold change in each analyte at the terminal time point for each macaque is shown. Data are represented as individual data points for each animal and time point.

The immune response during LASV infection is thought to play a critical role in disease outcome. Professional APCs are the primary target of the virus, and viral proteins are able to alter the maturation and activation of APCs, leading to diminished or ineffective immune responses, specifically those made by T-cells. In order to evaluate the kinetics of the immune response of NHPs infected with each virus, we examined changes in several groups of peripheral blood cells throughout the course of infection. While there were no clear differences in the number of CD4^+^ and CD8^+^ T-cells in the peripheral blood during infection between groups (Fig 10), there were some differences seen in certain T-cell subsets. CD4^+^ T-cells from animals infected with NML-33 had a higher proportion of naïve cells while those infected with NML-57 contained a higher proportion of memory cells overall during the terminal stage of disease (Fig 10A and 10B). A similar trend was observed whereby NML-33 animals had a higher proportion of naïve CD8^+^ T-cells while those infected with NML-57 had a higher proportion of memory cells (Fig 10C and 10D).

There were no clear differences in the proportion of activated CD69^+^ CD4^+^ T-cells between the groups. NML-57 infected animals had higher percentages of NK-like cells present compared to NML-33 and Josiah infected animals (Fig 7F). The induction of a strong Th1 and cytotoxic response, while possibly contributing to pathogenesis, may be critical for protection from lethal disease.

**Fig 11:**
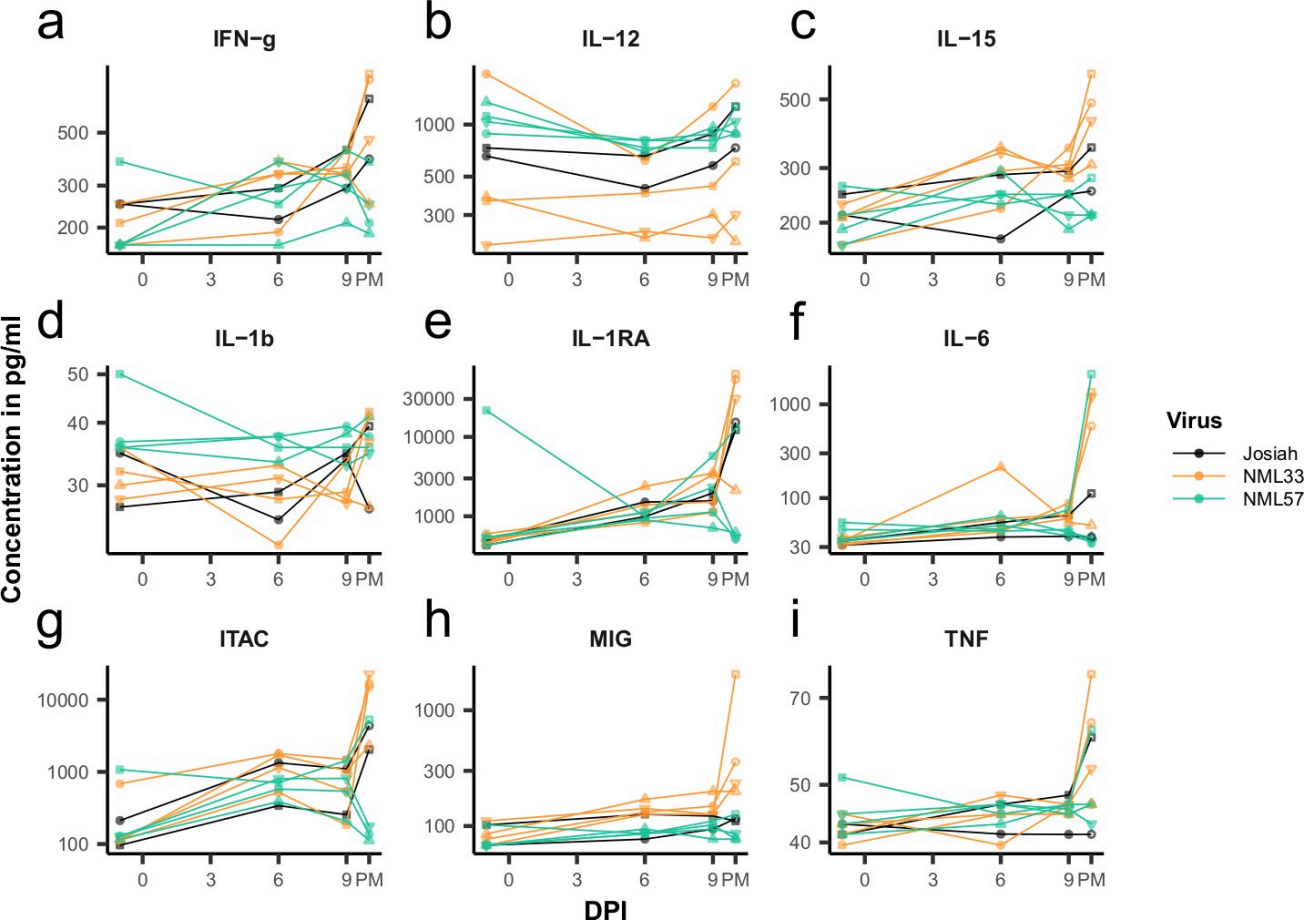
Cytokine and chemokine profiles during Lassa virus infection of macaques. Cytokine/Chemokine analysis was conducted using a 29-plex Luminex assay on each individual NHP at their respective time point. Shown are IFN-gamma (a), IL-12 (b), IL-15 (c), IL-1b (d), IL-1RA (e), IL-6 (f), ITAC (g), MIG (h), TNF (i).

In addition to immune cell phenotypes, we examined a number of cytokines, chemokines, and growth factors in the serum of infected animals using a 29-plex Luminex panel. Only a small number of cytokines and chemokines showed any difference throughout the course of infection between the groups of NHPs. Three out of four NML-33 animals at their terminal end point had cytokine profiles which clustered together with the response seen in one Josiah-infected animal, while the other NML-33 and Josiah animals showed closely related profiles (Figs 10F and 11). IL-6, MCP-1, IL-1RA, and I-TAC showed similar changes throughout the course of infection, particularly in the one Josiah and three NML-33 infected animals, which had increased levels of all four at their terminal end points (Fig 11). No other apparent trends differentiating the infecting strain of LASV throughout the course of infection were noticeable when comparing changes in cytokine or chemokine levels at different time points (Fig 10). NML-57 infection, despite leading to higher percentages of certain immune cell types in the peripheral blood, did not result in an increase in cytokine or chemokine levels in the serum. NML-33 infected animals did have higher levels of some cytokines compared to those infected with Josiah or NML-57. Pro-inflammatory cytokines IL-2, IFN-γ, IL-17A, and IL-6 were all increased to some degree at the end point for NML-33 infected NHPs (Figs 10 and 11). The chemokines I-TAC and MIP-1b, which are chemo-attractants that aid in T cell and macrophage trafficking, were also increased (Figs 10 and 11). Such chemokines may play a role in recruiting monocytes, macrophages and T cells into the tissues during inflammation, exacerbating pathogenesis.

## Discussion

Despite the 2018 outbreak isolates originating from northern Nigeria, significant phenotypic heterogeneity was observed in the three that were characterized in animal models of disease. Differences in the virulence of isolates from Liberia as well as Mali have previously been demonstrated in both guinea pigs and NHPs [18]. Three isolates (NML-33, -46 and -57) were tested for pathogenesis in strain 13 guinea pigs, with only the NML-33 (isolated from a fatal human case of LF) isolate demonstrating nearly uniform (5 of 6) lethal infection. Interestingly, only a single death was observed for NML-46 and NML-57 (isolated from a fatal and non-fatal case, respectively). When the NML-33 and NML-57 isolates were used to infect cynomolgus macaques, a similar trend was observed with all of the NML-33 NHPs (4/4) and only a single NML-57 NHP (1/4) succumbing to LASV disease. It is important to note that there were nearly identical pathogenic outcomes in both the strain 13 guinea pigs and NHPs for the isolates tested, lending to the relevance of the guinea pig model for future pathogenesis and screening of medical countermeasures. While we have identified a new lethal isolate (NML-33) for testing of LASV countermeasures, it cannot be underestimated that up to one-third of LASV survivors can develop permanent sensorineural hearing loss as well as other significant comorbidities as a result of LASV infection. The NML-57 isolate resulted in minimal lethality (1 of 4 animals, 25%). Although all NHPs in the group had detectable viral RNA in serial blood samples and demonstrated seroconversion by the end of the study, infectious virus was only detected in the single NHP which succumbed to infection. Throughout the study no overt clinical signs of disease were noted in the three surviving NHPs in the NML-57 challenge group though low levels of infectious virus from the liver and to a lesser extent lung specimens from two surviving animals at 42 dpi was observed (Figs 3B and 5B). Further studies with NML-57 are required to determine if it is a non-lethal LASV isolate in NHPs. Nevertheless, the importance of non-lethal models in the context of vaccine development and the prevention of sensorineural hearing loss as well as other comorbidities associated with LF, in addition to the possibility of viral persistence, will also play an important role in future vaccine development [24]. Furthermore, based on the parallels in our disease modelling efforts and the outcomes of the human cases from which these viruses were isolated, efforts should be focused on obtaining new LASV isolates from patients experiencing long-term sequelae of infection.

The most reliable biochemical indicator of disease outcome in the current study was a precipitous decline in albumin and total protein levels, both of which have been observed in other studies (Fig 7A and 7M) [18]. Excessive pro-inflammatory immune responses may exacerbate disease during LASV infection. Therefore the induction of regulatory T cells could be important for limiting any collateral damage done by excessive inflammatory responses. The induction of a more robust CD4+ and CD8+ memory T cell response in NML-57 infected animals may also be an indication of a more robust protective response as opposed to the NML-33 animals. The pan-T cell activation noted in these animals also suggests an overall inhibition of T cell activation in lethal infections with Josiah and NML-33, which has previously been shown in LASV infections (Fig 10) [18,25].

Several of the NML-33 infected NHPs had significantly increased pro-inflammatory cytokine levels whereas NML-57 NHPs did not (Fig 10). In a recent study with a Malian LASV isolate (Soromba-R), reduced pathogenesis and lethality with significant pulmonary involvement compared to the prototypical Josiah isolate was observed [18]. This was attributed to a strong TNF-α response, which may have increased the permeability of endothelial cells. However, we did not note any significant increases in TNF-α levels in any of the isolates tested despite significant pulmonary involvement and lethality with respect to the NML-33 isolate (Fig 10). The explicit observation of severe lung disease in the NML-33 isolate would lend itself to further characterization through blood gas monitoring and chest x-rays, neither of which were available at the time of the study.

More detailed studies of the immunobiology of not only LASV infection, but lethal versus non-lethal LASV isolates that are closely related could elucidate the role of humoral and cellular immunity in pathogenesis. It is striking that isolates from the same region and outbreak would result in starkly different pathological outcomes. This may be important in determining specific genomic regions of pathogenesis in the absence of robust reverse genetic systems, leading to new developments in drugs and therapeutics. A NHP model of LASV disease, which more accurately recapitulates the characteristic hallmarks of LF as observed in humans is of the utmost importance when testing novel medical countermeasures including vaccine candidates. However, the predictive value in modeling LF in strain 13 guinea pigs, in addition to practicality of use, make this model suitable for initial pre-clinical and pathogenesis studies of various LASV isolates. Our data highlights a much larger range of LASV disease pathogenesis in the broader context of disease outbreaks, which will be critical in identifying diagnostic indicators of disease and development of medical countermeasures.

## Methods

### Ethics statement

All animal studies were approved by the Animal Care Committee of the Canadian Science Center for Human and Animal Health (CSCHAH). Studies were conducted according to the Canadian Council on Animal Care (CCAC), in a CCAC approved facility by trained personnel. All procedures involving infectious LASV were performed in a BSL 4 laboratory of the Public Health Agency of Canada. When required, materials were inactivated according to approved internal procedures for subsequent analysis.

### Virus isolation

Serum samples were diluted 1:1000 in DMEM and used to inoculate nearly confluent monolayers of Vero cells. After 1 hr incubation at 37°C, inoculum was removed and replaced with DMEM supplemented with 2% FBS and further incubated until CPE exceeded 90%. Passage 1 (p1) supernatant was collected at 7 dpi, clarified and cyro-preserved for future studies. Titers were calculated using TCID_50_ methodologies

### Illumina sequencing

In order to sequence the new viruses, RNA was extracted from cell culture supernatant using the Qiamp Viral RNA mini kit (Qiagen) as per manufacturer’s instructions. The RNA was processed into double-stranded cDNA using the Maxima H minus dscDNA kit (ThermoFisher) following the manufacturer’s instructions. Libraries for Illumina sequencing were prepared using the Nextera DNA Flex kit (Illumina) using the maximum volume of input material and following the manufacturer’s instructions. Illumina sequencing (2 x 151 bp) was performed on a MiniSeq using a Mid-output Reagent pack (Illumina). Libraries for MinION sequencing were prepared using the PCR Barcoding Protocol (Oxford Nanopore Technologies Inc.) using the maximum input volume (dscDNA as input). Preprocessing for the Illumina reads was as follows: 1) adapter sequences and low quality bases (Q score below 25) were removed using *trim_galore* (v. 0.4.4_dev); 2) the reads were aligned to the genome of the African green monkey (*Chlorocebus sabaeus*; genome assembly GCF_000409795.2) using *bwa* [26] (v. 0.7.17-r1194-dirty); 3) unaligned reads were extracted using *gatk SamToFastq* (v. 4.0.4.0-37-g3189612-SNAPSHOT).

Preprocessing for the Nanopore reads consisted of the removal of host reads using *graphmap* [27] (v. 0.5.2) against the same African green monkey genome and extraction of unaligned reads using *gatk SamToFastq*. For each virus, *SPAdes* [28] (v. 3.12.0) was used to assemble both the Illumina and Nanopore reads using k-mer sizes of 77, 99, and 127. The resulting contigs were compared to the reference of all viral proteins from NCBI (downloaded on 27 August 2018) using *diamond* [29] (v. 0.9.22.123). Contigs containing LASV sequences (usually only 2, one for L and one for S) were blasted on the NCBI website using blastn to find the closest known virus. The S and L sequences from the closest virus were used in *nucmer* [30] (v. 4.0.0beta2) to find the limits and orientations of all fragments. A custom python script was used to trim the ends of the new sequences and organize them in the same orientation as the reference viruses. Annotations were transferred from the closest reference virus using *RATT* [31] (from PAGIT v1.64).

### Phylogenetic analysis

Sequences for the S segments of 287 LASV isolates were downloaded from GenBank, along with the sequence to Mopeia virus strain AN 21366 (accession # M33879.1). (List of accession numbers in S1 Appendix). The sequences of the five new strains were aligned to the references using *MAFFT* (v. 7.310). The output was converted to nexus format using *seqmagick* (v. v0.7.0+7.g1642bb8). The year of isolation of all the references were obtained from the GenBank records or from the original publications. BEAST v2.5.1 (with BEAGLE library v 3.0.1) [32–34] was used to run a phylogenetic reconstruction assuming a Coalescent Constant Population model with a relaxed lognormal clock and a generalized time-reversible substitution model. (BEAST XML configuration file in S2 Appendix). Three chains of BEAST2 were run for 100 million iterations and the last 75 million were used for downstream analysis (after confirming convergence using Tracer v. 1.7.1). The trees were down sampled to a total of 20 000 trees for downstream analysis and 101 trees for plotting using the *ggtree* R package [35,36] (v. 2.0.0). TreeAnnotator was used to analyze the trees to provide a maximum clade credibility tree with 95% HPDIs.

### Animal studies

Strain 13 guinea pigs (*Cavia porcellus*, 0.8 to 1 kg, mixed gender) were sourced from the in-house breeding colony at Rocky Mountain Laboratories (National Institutes of Health, National Institute of Allergy and Infectious Diseases). All guinea pigs were outfitted with a subcutaneous electronic transponder temperature chip (Bio Medic Data Systems Inc.) prior to being transported to a BSL4 laboratory. Groups of 6 guinea pigs (4 male, 2 female) were infected with 1x10^4^ TCID_50_ of each Nigerian isolate (NML-33, NML-46, or NML-57) by the intraperitoneal route. Guinea pigs were housed in ferret isolators that provide negative pressure and HEPA- filtered containment and monitored daily for signs of disease.

Ten female cynomolgus macaques (*Macaca fascicularis*, 3 to 4 kg) were procured from approved sources and acclimated for at least 7 days prior to the study. Animals were challenged by intramuscular injection with 1x10^4^ TCID_50_ of either LASV Josiah (n = 2) or the Nigerian isolates NML-33 (n = 4) and NML-57 (n = 4). Animals were monitored twice daily for signs of disease and scored based on an endpoint chart designed for LASV disease. Scoring criteria was based on numerical values assigned to the severity of specific visual indicators including posture, respiration rate, feces/urine output and consistency, food and water intake, general attitude and recumbency, as well as signs of cyanosis, petechiation, discharges and hemorrhage. Euthanasia decisions were made based on evaluations of clinical signs and the overall status of each specific animal by trained personnel. Blood and serum were collected at scheduled times before and after challenge (days -7, 0, 1, 3, 6, 9, 13, 16, 22, and 29 with respect to infection) for analysis, which included hematology, clinical chemistry, flow cytometry cytokine analysis, serology and viremia. Rectal temperature and body weights were also monitored concurrently with blood draws. Upon humane euthanasia, tissues (liver, lung, spleen, heart, and kidney) were colleted.

### Hematology, biochemistry and coagulation

Hematological analysis was conducted on EDTA-treated blood samples using a VetScan HM5 (Abaxis), while serum biochemistry analysis was conducted using a VetScan VS2 (Abaxis) with comprehensive diagnostic profile discs. Citrate plasma was also collected in order to measure coagulation parameters which included: fibrinogen, aPTT, thrombin, PT, Protein C, and Protein S. A Start4 instrument from Diagnostica Stago was used to perform all assays using the manufacturer’s instructions.

### Molecular detection of LASV RNA

LASV RNA was extracted from whole blood using a QIAamp Viral RNA mini kit (Qiagen) according to the manufacturer’s instructions. A 25μl reaction was set up using 1-step RT-PCR master mix kit (Qiagen) and with amplification and detection on a QuantStudio Real-Time PCR system (Qiagen) according to the manufacturer’s specifications. The following forward (5’-ATGGCTTGTTTGTTGAAGTCRAA-3’) and reverse (5’-TGACCAGGTGGATGCTAATTGA-3’) primers in conjunction with probe (5-FAMCATGTCACA-ZEN-AAATTCTTCATCGTGCTTCTCA-IABK-3) targeting a conserved region of the LASV glycoprotein were used. A standard curve estimating the number of genomic copies per milliliter of whole blood was established using a plasmid containing the full LASV S segment.

### Infectious titrations (TCID_50_)

96-well tissue culture plates were seeded with VeroE6 cells in order to become 70–80% confluent within 24 hr. Liver, lung, spleen, heart and kidney samples were homogenized in 1 ml of DMEM using a TissueLyserII (Qiagen). The homogenates were centrifuged at 10,000×g for 10 min. Clarified homogenates or sera were ten-fold serial diluted in DMEM containing 2% FBS and 100 μl of each dilution in triplicate were added per well. Cells were then incubated for 7–10 days at 37°C with 5% CO_2_. Cells were assessed for virus-induced cytopathic effect and the tissue culture infectious dose (TCID_50_) was calculated by the Spearman-Karber method.

### Cytokine milliplex assay

Expression levels of cytokines, chemokines and growth factors were assessed in NHP serum samples by using a 29-plex NHP cytokine panel according to the manufacturer’s recommendation (Thermo Fisher). The serum samples were γ-irradiated (5 Mrad, Cobalt-60 source) and the concentrations of cytokines and chemokines were determined in the NHP samples using the Luminex MAGPIX system (Thermo Fisher). The following cytokines and chemokines were targeted for the study: Interleukin-1β (IL-1β), IL-1 receptor antagonist (IL-1RA), IL-2, IL-4, IL-5, IL-6, IL-8, IL-10, IL-12, IL-15, IL-17, Eotaxin, basic fibroblast growth factor (FGF-basic), granulocyte colony-stimulating factor (G-CSF), granulocyte macrophage colony-stimulating factor (GM-CSF), IFN-γ, IFN-inducible protein 10 (IP-10), monocyte chemoattractant protein-1 (MCP-1), macrophage inflammatory protein-1α (MIP-1α), MIP-1β, regulated-on activation normal T-cell expressed and secreted (RANTES), tumor necrosis factor-α (TNF-α), vascular endothelial growth factor (VEGF), hepatocyte growth factor (HGF), monokine induced by interferon-gamma (MIG or CXCL9), interferon-inducible T cell alpha chemoattractant (I-TAC), macrophage-derived chemokine (MDC or CCL22), macrophage migration inhibitory factor (MIF) and epidermal growth factor (EGF). Briefly, 25 μl of 1x anti-cytokine antibody-coupled beads were added to the 96- well flat bottom plate, inserted on the magnetic separator for 30–60 seconds followed by a wash two times with wash buffer (Thermo Fisher, wash procedure). 50 μl of irradiated NHP serum was diluted to 1:4 and incubated with incubation buffer for 2 hr at room temperature. The serum-bead complexes were washed two times with wash buffer using the magnetic separator wash procedure (Thermo Fisher). The complexes were further incubated with a 1x biotinylated detector antibody for 1 hr, followed by 1x streptavidin-RPE solution incubation for 30 min. The plate wells were washed a final three times with wash buffer and the complexes were re-suspended in 150 μl of wash solution for acquisition of the bead count (50 beads) in the Luminex MAGPIX instrument. The final concentration of each analyte was expressed in pg/ml using the mean fluorescence intensity (MFI) rate.

### Flow cytometry

Flow cytometry was performed at the specified time points using the following antibody cocktail (all from BD Biosciences): CD45 FITC (10μl/test), CD25 PE (20μl/test), CD4 PerCP-Cy5.5 (20μl/test; clone L200), CD69 PC7 (5μl/test), CD8 APC-H7 (5μl/test; clone RPA-T8), CD3 Alx700 (1μg; clone SP34-2), CD16 Pac Blue (5μl/test), CD20 APC (0.2μg), CCR7 BV711 (0.5ug; from BioLegend; clone), CD45RA PE-CF594 (5μl/test), CD14 BV650 (5μl/test). For each time point, 100μl of whole blood was collected and mixed with 5μl of TruStain FcX (BioLegend) for 10 min at room temperature (RT). The antibody cocktail was added to each sample and incubated for 20 min at RT. Following the incubation each sample was lysed with 1x FACS lyse solution, spun down, and the supernatant was decanted. The samples were washed with 2 ml of PBS, spun down and decanted again. Samples were inactivated using a standard protocol for removal of cells from our BSL-4, involving two incubations of 30 min in at least 200 μl of BD Cytofix/Cytoperm per million cells (based on HM5 white blood cell count). To prepare each blood collection for flow cytometry, inactivated samples were transferred to a 5ml polystyrene round-bottom tube with 1ml PBS. Samples were spun at 500xg for 8 min, decanted, and cells were re-suspended in 200ul of PBS before being run on the BD LSR II (BD Biosciences). All analysis was performed using Flowjo vX.3 (Treestar).

### Histopathology

Immediately post-collection, tissue specimens were submerged in 10% Neutral Buffered Formalin and fixed for 28 days. Tissues were placed in cassettes and processed with a Sakura VIP-6 Tissue Tek, on a 12-hr automated schedule, using a graded series of ethanol, xylene, and PureAffin. Embedded tissues were sectioned at 5μm and dried overnight at 42°C prior to staining with hematoxylin and eosin (H&E) according to standard histopathological methods.

### Serology

In order to determine seroconversion in surviving animals (guinea pigs and NHPs) a commercial Pan-Lassa NP IgG ELISA (Zalgen Labs) was utilized with modifications. Briefly, guinea pig and NHP serum samples were diluted 1:100 and incubated for 30 min at RT on pre-coated ELISA plates pre-coated with recombinant LASV nucleoprotein. The plates were washed four times with the kit-provided wash buffer. The kit provided anti-human specific IgG secondary antibody was used for NHP samples whereas a secondary goat anti-guinea pig specific IgG (H&L)-HRP (SeraCare) antibody was used for guinea pig samples at a dilution of 1:1000. Plates were incubated for another 30 min and then washed 4 times. The kit-provided one-component substrate was added and incubated for 10 min. The reaction was stopped using 0.36 N sulfuric acid and the optical density of samples read at 405 nm.

## Supporting information

S1 Appendix Accession numbers for sequences utilized in phylogenetic analysis in Fig 1. (CSV)

S2 Appendix BEAST xml file (for version >2.5) for the phylogenetic analysis in Fig 1. (XML)
